# Generative AI and knowledge management in health education: a cross-sectional study of students’ digital literacy and attitudes from a developing-country context

**DOI:** 10.1186/s12909-026-09373-7

**Published:** 2026-05-13

**Authors:** Ermira Krasniqi, Adea Kasumi, Arjeta Kuçi, Elda Murić, Elsa Ahmeti, Laura Naka, Arben Boshnjaku

**Affiliations:** 1 Faculty of Pharmacy, Alma Mater Europaea Campus College Rezonanca, Prishtina, Kosovo; 2 Faculty of Medicine, University “Fehmi Agani” in Gjakova, Ismail Qemali, n.n., Gjakova, 50000 Kosovo; 3 Faculty of Education, University “Fehmi Agani” in Gjakova, Gjakova, Kosovo

**Keywords:** Artificial intelligence, Higher education, Health education, Digital literacy, Generative AI, Developing countries

## Abstract

**Background:**

Generative artificial intelligence (AI) is rapidly transforming higher education and digital learning. However, a scarcity of information remains regarding the students’ perspectives on AI’s role in education. We investigated AI-related knowledge, use, and attitudes among entry-level healthcare students in Kosovo.

**Methods:**

Cross-sectional online survey (June–August 2025) of entry-level students from two institutions (≈ 700 invited; *N* = 302; response 43.1%). Analyses used χ² with Cramér’s V, and Kruskal–Wallis with ε²; α = 0.05.

**Results:**

ChatGPT was reported as the primary AI instrument (84.2%). 74.8% of participants perceived AI integration positively, 77.2% supported curricular integration, and its ability to facilitate access to information was 91.4%. Interest in AI training was as high as 71.2%, whereas major concerns included error risks (29.5%) and reduced creativity (29.0%). Program differences were observed for faculty use of AI-assisted materials (*p* = 0.006; Cramér’s V = 0.20), AI-training opportunities (*p* = 0.037; V = 0.17), and institutional AI-tool provision (*p* = 0.032; V = 0.151); self-rated AI knowledge did not differ (*p* = 0.274; ε²=0.003).

**Conclusions:**

Findings indicate general positivity towards AI, alongside concerns regarding unequal access and limited formal training. These findings underscore the importance of integrating generative AI within e-learning strategies, strengthening students’ digital literacy, and ensuring institutional support in developing country settings.

## Introduction

The Fourth Industrial Revolution, or Industry 4.0, has accelerated the integration of advanced technologies in sectors such as education, healthcare, and scientific research [1, 2]. Amongst these, artificial intelligence (AI) has emerged as a transformative force, enabling data-driven decision-making, personalized learning, and automation across complex systems [3]. In health education, AI is driving curricular reform globally, reshaping teaching methods, influencing resource distribution, and expanding learning opportunities beyond traditional time and place constraints [4]. Particularly, generative AI through large language models is directly influencing not only healthcare education but also cognitive processes such as learning, reasoning, and creativity, making its implications equally relevant to health, psychology, and education research.

Although institutional and faculty-level responses to AI adoption have been widely discussed, students’ perspectives remain underexplored to date. Understanding how students perceive, use, and are influenced by AI is critical for determining whether these technologies enhance learning or contribute to disengagement. In this modern digital landscape, competencies like Digital Health Literacy (DHL), the ability to seek, evaluate, and apply digital health information, and the Evidence-Based Practice (EBP), which integrates clinical expertise with the best available evidence, are essential. Digital literacy, particularly DHL, plays a critical role in shaping how students engage with generative AI, influencing not only their ability to access and evaluate AI-generated information but also the depth and quality of its use in learning contexts [5, 6]. In this context, effective engagement with generative AI requires the ability to critically evaluate and apply digital information, a process closely related to digital health literacy and supported by structured training and curricular integration [7, 8]. It is argued that failing to integrate DHL and EBP into healthcare education risks widening disparities and undermining public health efforts [6, 9]. Considering how studies from high-income countries are predominantly dominating the discourse, there seems to be a lack of empirical evidence from developing and transitional contexts, where digital literacy, infrastructure, and perceived institutional readiness vary widely. Identifying students’ voices and beliefs in such contexts is essential to avoid widening global inequities in AI integration.

With AI increasingly influencing healthcare, health education must evolve to balance technical competences with ethical awareness. Health-care students are encountering the application of AI in academic, clinical, and research settings, yet concerns persist about challenges including accuracy, bias, ethical use, and academic integrity [10]. These opportunities and challenges emphasize the immediate need to examine how future healthcare professionals, particularly those residing in low-resource settings, are prepared to engage with the AI responsibly. Nonetheless, despite the growing exposure to AI and its countless possibilities, limited research exists on students’ knowledge, usage, and perceptions of AI, particularly in low- and middle-income countries.

**Fig. 1:**
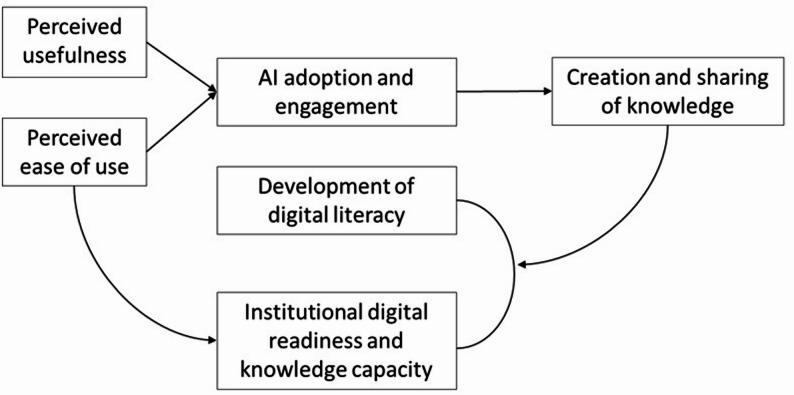
Conceptual framework linking generative AI, technology acceptance, and knowledge management in entry-level health education

While high-income countries are actively integrating AI into health education through structured interdisciplinary programs [8], developing countries face significant challenges. These include limited digital infrastructure and access to resources, and a lack of formal training opportunities [11]. Similar barriers have been noted in other developing countries like the case of Egypt where financial constraints, limited technology access, and inadequate training were reported [12]. In Nigeria and India, students show both optimism and concern; in Kerala, for instance 57.2% viewed AI as reducing medical errors, while 69.2% feared it might erode medicine’s humanistic aspect [13]. Evidence from nearby Slovenia, in a broader educational context, highlights a comparable digital divide: students are receptive to AI but adoption depends on institutional support, infrastructure, and training [14]. Research from Japan further shows that well-structured online programs can even outperform face-to-face models in building collaboration and readiness [15], emphasizing the role of program design in shaping outcomes. Kosovo, like many developing nations, is in the early stages of integrating AI into healthcare education, with limited data on how health students engage with generative AI or perceive its educational value and institutional support. This study is conceptually informed by the Technology Acceptance Model (TAM) which considers the usefulness and ease of use to determine the technology adoption, and in Connectivist learning theory that considers learning as both creation and navigation of knowledge networks. Importantly, these frameworks are used to support the interpretation of findings and to situate the study within existing theoretical perspectives, rather than to serve as empirically testable models within the current analysis [16]. As such, the framework serves as a conceptual supplement rather than a basis for causal modelling or predictive statistical analysis. Such frameworks guide the interpretation of generative AI adoption as both technological and pedagogical phenomenon. It should be noted that from the perspective of knowledge-management, generative AI may provide a potential infrastructure for creating, organizing, and disseminating knowledge. The understanding of how students interact with such tools might inform institutional strategies for fostering digital learning ecosystems. The conceptual relationships explored in this study are illustrated in Fig. 1.

In addition, differences across healthcare disciplines may further influence AI adoption and use, as educational structures, clinical exposure, and technological integration vary between programs. For instance, disciplines with stronger emphasis on evidence-based decision-making or data-driven practice may engage differently with generative AI compared to those with more patient-centered or manual therapeutic approaches. Empirical evidence supports this variability; for example, Teng et al. (2022) found that students’ understanding of the ethical implications of AI differed across professions, with physiotherapy and dentistry students demonstrating greater awareness than those in medicine, nursing, and midwifery [17]. Emerging evidence indicates that disciplinary context and task requirements significantly shape AI usage patterns, perceived barriers, and educational needs [18, 19]. Therefore, comparing programs provides insight into how disciplinary context shapes AI use, training opportunities, and perceptions.

The rationale for comparing different health-related disciplines originates from the distinct pedagogical requirements and clinical workflows inherent to each profession. While pharmacy education may leverage AI for pharmacological precision and drug-interaction modeling, nursing and physiotherapy curricula often emphasize clinical reasoning and patient-provider communication. Therefore, understanding these disciplinary nuances is essential for developing tailored AI integration strategies. Furthermore, the rapid adoption of generative AI necessitates an evolution in DHL frameworks. In fact, DHL must now transcend basic information retrieval to include the critical appraisal of AI-generated content, as a student’s ability to detect algorithmic bias or “hallucinations” becomes a core competency in modern health education.

Therefore, this study aims to evaluate students’ knowledge of AI, perceptions, and perceived institutional readiness for AI integration in higher education, using healthcare programs in Kosovo as a case study. By identifying key trends, opportunities, and concerns, the research seeks to inform the development of context-sensitive educational strategies, support faculty and curriculum designers in addressing student needs, and contribute to broader efforts toward equitable and responsible AI integration in undergraduate health education. The main objective is to compare AI knowledge, use, and attitudes across programs (pharmacy, nursing, physiotherapy, public health) and to examine perceived institutional and faculty readiness among entry-level students in Kosovo.

## Materials and methods

### Participants

This cross-sectional study used a convenient sample of students from two higher education institutions in Kosovo (public University “Fehmi Agani” in Gjakova and private AMECC Rezonanca college) that provide entry level education in different study disciplines. The survey was conducted between June and August of 2025, while administered digitally via Google Forms and disseminated to participants through various platforms, including email, Facebook, WhatsApp, and the Students Forum WhatsApp group. In order to maintain data integrity, the form was configured to permit only a single submission per participant. The survey link was distributed to approximately 700 students across both institutions (340 from UFAGJ and 360 from AMECC Rezonanca), of whom 302 students completing the questionnaire (response rate = 43.1%). No cases were excluded from the analysis. The survey was completed by 302 students from four programs (pharmacy, nursing, physiotherapy and public health). While the study focused on healthcare-related programs, findings contribute broadly to understanding AI adoption in higher education settings in developing countries. Eligibility was limited to full-time undergraduate students enrolled in entry-level healthcare programs. Pilot participants (*n* = 10) were excluded from the analysis. Participation was entirely voluntary with no incentives offered and a complete anonymity was ensured.

A permission to conduct the research was granted by the Ethical Committee of the Faculty of Medicine, University “Fehmi Agani” in Gjakova (protocol no. 006/364, date 11.06.2025).

### Assessment instrument

For collecting data this study used a questionnaire developed by Risana VU and colleagues [20] to assess the knowledge, application, and perspectives of higher education students regarding AI. From the original questionnaire [20], the following questions were excluded / adopted “Age groups” (we required the exact age of participant), “domicile” (deleted).

The process of translation and adjustment to Albanian language consisted of four steps:

- Step 1 - the questionnaire was translated from English to Albanian by a healthcare expert (Version 1).
- Step 2 - two healthcare experts analysed the translation once again and decided on the version to proceed (Version 2).
- Step 3 - questionnaire was blindly translated backwards from Albanian to English by a university professor of English language (Version 3).
- Step 4 - the two experts involved in step 2 together with the expert who translated the questionnaire compared the differences between the original version and the backward translated one, and consensually decided on the final version (Version 4).

This instrument was developed by incorporating findings from extant research and was structured into four subsections: (a) sociodemographic, (b) awareness, applications, and benefits of AI, (c) experiences with AI, and (d) viewpoints and attitudes towards AI ^20^. The sections including (i) sociodemographic data, including age, gender, level of education, and educational institution; (ii) knowledge about artificial intelligence, including questions regarding basic knowledge, current uses, and potential benefits of AI in education; (iii) personal experiences with AI in education, aiming to understand whether and how students had experienced the use of AI during their studies, and (iv) attitudes and opinions toward AI, perceptions, and willingness to use this technology in the future. The final instrument comprised 26 items: knowledge (7 items), use/experience (5 items), and attitudes (8 items). In this study, ‘AI use’ refers to self-reported engagement with generative AI tools (e.g., ChatGPT) for academic-related purposes, including information retrieval and concept clarifications. The questionnaire did not differentiate between depth or mode of use (e.g., passive vs. interactive engagement), which should be considered when interpreting findings, as this distinction may influence how AI integration is understood in educational contexts. Content validity was ensured through expert panel review during translation and adaptation. Before the main data collection, a pilot (*n* = 10) confirmed clarity and timing (~ 12 min; α = 0.71 for pilot items). However, in the full dataset, internal consistency was lower (see Results), so we analyzed items individually rather than forming subscale totals. Despite using a previously validated instrument, the lower internal consistency may reflect several factors. The questionnaire captures multiple item-level responses (knowledge, use/experience, and attitudes), which may not be unidimensional. In addition, cross-cultural adaptation may have influenced item interpretation, and the emerging nature of generative AI may have resulted in heterogeneous student experiences, reducing inter-item correlations. Given ordinal response formats, α 95% CIs were estimated by bootstrap, with ordinal reliability/ω is provided in Supplementary sensitivity analyses (if computed). Pilot participants were excluded from the main study. The finalized version contained 26 questions. Likert items used 5-point scales (1 = strongly disagree to 5 = strongly agree). Subsections comprised: knowledge (7 items), use/experience (5 items), attitudes (8 items).

### Statistical analysis

All statistical analyses were performed using IBM SPSS version 27 (SPSS, Inc., Chicago, IL, United States). Significance level was set at *p* < 0.05. Data were presented as continuous variables (in the form of mean and standard deviation) or categorical variables (relative frequencies). All variables were analysed at the item level. Likert-scale responses (1–5) were treated as ordinal variables and summarized using mean ± SD, while binary and categorical variables (e.g., yes/no or multiple-choice responses) were analyzed using frequency distributions and percentages. No composite scores were calculated due to the low internal consistency of the questionnaire; therefore, all results reflect individual item responses.

Continuous and ordinal variables (Likert-scale responses) were assessed for normality with the Shapiro–Wilk test and for homogeneity of variance with Levene’s test. As assumptions were not met, nonparametric Kruskal–Wallis tests were conducted to compare study programs, with ε² reported as the effect size. When significant, pairwise comparisons were examined using Mann–Whitney U tests with Bonferroni correction. Categorical variables were analysed with Chi-square tests of independence, with Cramér’s V reported as the effect size. All analyses were conducted on complete cases, with no presented missing data. Effect sizes were interpreted following Cohen’s guidelines [21], where Cramér’s V values of 0.10, 0.30, and 0.50 represent small, medium, and large effects, respectively.

Reliability analysis was conducted using Cronbach’s α to assess the internal consistency of the questionnaire subsections (knowledge, use/experience, and attitudes) and the combined instrument. Confidence intervals (95% CI) for α were estimated using bootstrapping with 1,000 resamples. For multi-choice items, responses were expanded into binary indicators before analysis.

## Results

### Sociodemographic characteristics of participants

302 students participated in this study. Table 1 provides descriptive data of participants. The majority of the participants were biological women (68.5%), while biological men constituted 31.5% of the sample. Mean age of participants was 21.4 ± 3.8 years, corresponding to the typical university student age, whereas the dispersal between academic years was as follows: 13.2% from 1st year, 30.5% from second year and 56.3% from the final third year. In terms of academic background, students were distributed across four different programs, from pharmacy (36.1%), nursing (33.1%), physiotherapy (16.2%), and public health (14.6%) programs (Table 1). Furthermore, students differed significantly (data not shown in tables) between study programs (*p* = 0.004, Cramér’s V = 0.49), as well as between study program and year of study (*p* < 0.001). Pharmacy students were predominantly in their final year, whereas most public health students were in their 1st year. On the other hand, nursing and physiotherapy students were generally distributed within the 2nd and 3rd years.

**Table 1:** Sociodemographic characteristics of the participants

Category	Frequency (*n* = 302)
Gender	
Women (n, (%))	207 (68.5)
Men (n, (%))	95 (31.5)
Age (y, mean ± SD)	21.4 ± 3.8
Pharmacy (y, mean ± SD)	21.9 ± 3.7
Nursing (y, mean ± SD)	20.9 ± 2.3
Physiotherapy (y, mean ± SD)	22.4 ± 6.3
Public Health (y, mean ± SD)	19.9 ± 2.4
Study program	
Pharmacy (n, (%))	109 (36.1)
Nursing (n, (%))	100 (33.1)
Physiotherapy (n, (%))	49 (16.2)
Public Health (n, (%))	44 (14.6)
Year of study	
1st (n, (%))	40 (13.2)
2nd (n, (%))	92 (30.5)
3rd (n, (%))	170 (56.3)

*Abbreviations: y* year, *n* number, *SD* standard deviation

### Reliability analysis

Internal consistency for the overall questionnaire (*n* = 302) was modest (Cronbach’s α = 0.202, 95% CI: 0.108–0.417). Subscale reliability ranged from poor to moderate (α = 0.42–0.65). Subsection analysis indicated limited internal consistency across subsections: Knowledge (α = 0.298, 95% CI: 0.137–0.435), Use/Experience (α = − 0.020, 95% CI − 0.342–0.146), and Attitudes (α = 0.012, 95% CI: 0.145–0.162). Given the suboptimal internal consistency of the instrument, findings should be interpreted at the level of individual items rather than as reflecting underlying constructs such as AI related knowledge, use or attitudes.

### Knowledge and experience with AI in education

Participants’ self-rated knowledge on AI did not differ significantly between the study programs (*p* = 0.274, ε² = 0.003), indicating rather similar levels of perceived knowledge. When assessing participants’ “knowledge of benefits from AI integration in curricula” and the “knowledge of current AI integration in curricula”, non-statistically significant differences between the programs (*p* = 0.401 and *p* = 0.759, respectively) could be observed either. With the effect sizes being small, program affiliations do not explain variances in students’ ratings.

Regarding the use of AI in academia, significant differences were found in all variables of students from different programs. There was a significant association between study programs on whether students being informed on professors using AI-prepared materials (*p* = 0.006) with a Cramér’s V of 0.2 indicating a small-to-moderate association. Similar significant outcomes (*p* = 0.037) and association (Cramér’s V of 0.17) were observed on whether having received training on AI integration in teaching, thus suggesting unequal opportunities for AI-related professional development across programs. Institutional provision of AI tools was the last variable to provide statistically significant differences between groups (*p* < 0.05), though with a small association (Cramér’s V of 0.151). In this context, nursing students reported the highest institutional access (33.0%), followed by pharmacy (25.7%), physiotherapy (16.3%) and public health (9.1%) students. To better understand the reasons behind the statistically significant differences observed between student groups in the three last questions from Table 2, a comparison was conducted between pharmacy students as representing one institution and the three other programs that were coming from the other higher education institution (data not added in the table). In the three cases, no statistically significant differences could be detected (*p* > 0.05).

**Table 2:** Participants AI knowledge and use by study program

	Total	Pharmacy	Nursing	Physiotherapy	Public health	*P* value	Effect size
AI knowledge (1–5)*	3.6 ± 0.9	3.5 ± 1.0	3.7 ± 0.7	3.8 ± 0.8	3.6 ± 1.1	*p* = 0.274	ε² = 0.003
Knowledge of benefits from AI integration in curricula (1–5)	3.8 ± 0.9	3.7 ± 1.0	3.7 ± 0.8	3.9 ± 0.7	3.8 ± 1.1	*p* = 0.401	ε² < 0.001
Knowledge of current AI use in curricula (1–5)	3.8 ± 0.9	3.8 ± 1.0	3.8 ± 0.8	3.9 ± 0.8	3.8 ± 1.1	*P* = 0.759	ε² < 0.001
Teachers informed on using AI-assisted teaching material (yes, n (%))	97 (32.1)	32 (29.4)	32 (32.0)	25 (51.0)	8 (18.2)	*p* = 0.006	Cramér’s V = 0.20
Received formal training on AI integration in teaching (yes, (%))	65 (21.5)	23 (21.1)	30 (30.0)	7 (14.3)	5 (11.4)	*p* = 0.037	Cramér’s V = 0.17
Institution provides AI tools (yes, n (%))	73 (24.2)	28 (25.7)	33 (33.0)	8 (16.3)	4 (9.1)	*p* = 0.032	Cramér’s V = 0.151

All variables represent individual questionnaire items and composite scores were not calculated. Values are mean ± SD or n (%). Tests: χ² with Cramér’s V for categorical; Kruskal–Wallis with ε² for continuous

*Abbreviations: AI* artificial intelligence

* scale 1–5 (minimum to maximum)

Amongst students reporting to have received formal training on AI integration in teaching (65 individuals or 21.5% of total participants), the major sources of AI training and information included internet as the primary source (28 or 9.3%), followed by seminars or presentations (14 or 4.6%), informal learning from peers or family (10 or 3.3%) and free webinars (8 or 2.6%). Formal or structured sources such as paid courses were the least common (4 or 1.3%).

**Fig. 2:**
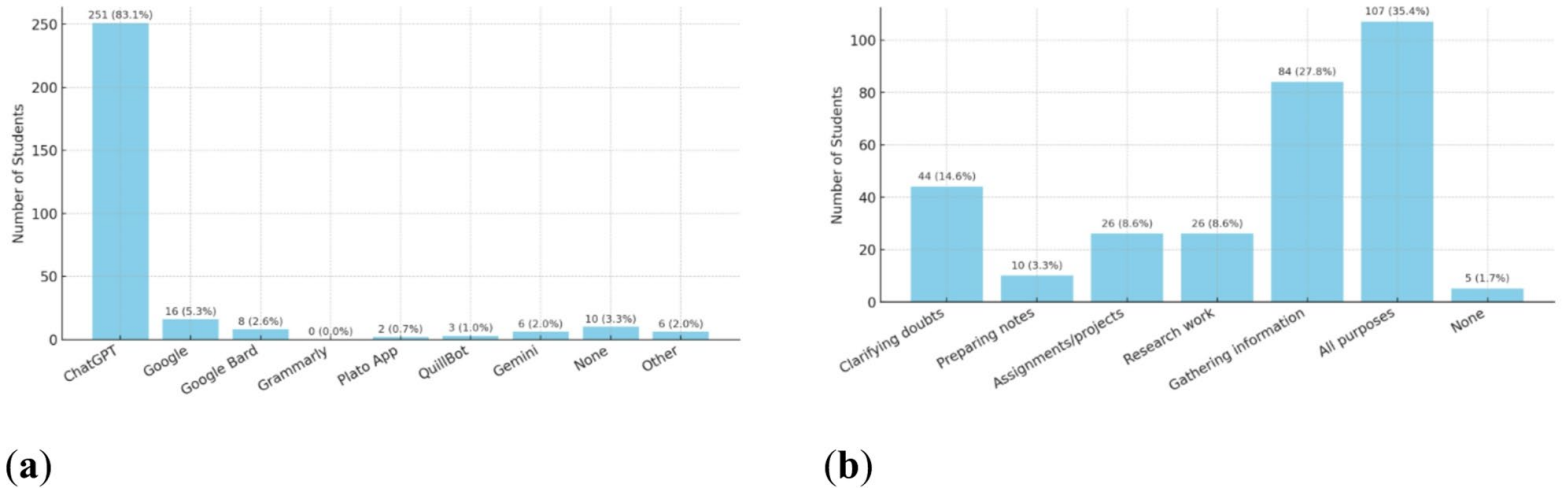
Patterns of AI tool use and the purposes in student learning: (**a**) Distribution of AI tools that are used by students; (**b**) main purposes of AI use

ChatGPT was reported to be used as the primary AI tool by the majority of participants (84.2%), with the rest of the students relying on Google (5.4%), Google Bard (2.7%), or other platforms. Interestingly, only 3.4% of students indicated not using any AI tools (Fig. 1). Furthermore, students reported diverse and unspecified purposes for using AI (35.8% reported “all purposes”), followed by gathering information (28.1%), and clarifying doubts (14.7%). A rather small percentage of students reported using AI for assignments/projects or research work (8.7% in both cases), and only 3.7% reported not using AI at all. Patterns of AI tool use and purposes of AI-assisted learning are presented in Fig. 2.

When comparing participants’ responses as separated by biological gender, no significant differences were observed across AI knowledge, experiences or attitudes, except for institutional provision of AI tools, where men reported slightly higher access than women (*p* = 0.013, Cramér’s V = 0.17).

### Attitudes on AI

Overall, 74.8% of students perceived AI integration positively, though differences between study programs were notable (highest in physiotherapy and pharmacy, lowest in public health). Table 3 shows the attitudes of participants regarding AI in total and separated into education programs. This perceived impact of AI integration varied significantly across study programs (*p* < 0.001, Cramér’s V = 0.21). Physiotherapy and pharmacy students reported predominantly positive effects (85.7% and 83.5%, respectively), followed by nursing (68.0%) and public health (56.8) students. Furthermore, the fact that association observed between study program and perceived impact of AI integration was statistically significant (*p* < 0.001) with a Cramér’s V of 0.21 indicating a small-to-moderate effect size, suggests that program affiliation explains some but not all of the variance in students’ attitudes.

**Table 3:** Attitudes toward AI integration among healthcare students in Kosovo, stratified by study program

	Total	Pharmacy	Nursing	Physiotherapy	Public health	*P* value	Effect size
The perceived impact of AI integration (positive, *n* (%))	226 (74.8)	91 (83.5)	68 (68.0)	42 (85.7)	25 (56.8)	*p* < 0.001	Cramér’s V = 0.21
AI facilitates access to information (yes, n(%))	276 (91.4)	103 (94.5)	89 (89.0)	46 (93.9)	38 (86.4)	*p* = 0.277	Cramér’s V = 0.11
Importance of AI integration in curricula (yes, n(%))	233 (77.2)	88 (80.7)	84 (84.0)	40 (81.6)	21 (47.7)	*p* < 0.001	Cramér’s V = 0.29
Students should develop AI-based knowledge and skills (yes, n(%))	213 (70.5)	76 (69.7)	75 (75.0)	34 (69.4)	28 (63.6)	*p* = 0.566	Cramér’s V = 0.08
Interest in AI training workshops (yes, n (%))	215 (71.2)	78 (71.6)	77 (77.0)	37 (75.5)	23 (52.3)	*p* = 0.021	Cramér’s V = 0.18
AI could substitute healthcare professionals (yes, n(%))	64 (21.2)	28 (25.7)	19 (19.0)	5 (10.2)	12 (27.3)	*p* = 0.106	Cramér’s V = 0.14

Values are mean ± SD or n (%)

Tests: χ² with Cramér’s V for categorical; Kruskal–Wallis with ε² for continuous

*Abbreviations* AI, artificial intelligence

* scale 1–5 (minimum to maximum)

One point of interest was the agreement that AI facilitates access to information being overwhelmingly high across all programs (> 86%) and did not differ significantly in between (*p* = 0.277, Cramér’s V = 0.11). Pharmacy (95%) and Physiotherapy (94%) students reported the highest agreement, with Public Health students somewhat less unanimous (86%), though very high. Notwithstanding this, opinion on whether AI should be integrated into the curriculum of educational programs of higher education systems differed significantly between study programs (*p* < 0.001, Cramér’s V = 0.29). The absolute majority of nursing (84.0%), physiotherapy (81.6%) and pharmacy (81%) students were positive, with the majority of public health students thinking the opposite.

As anticipated, the majority of participants agreed that students should develop AI-related knowledge and skills (63.6–75.0%), with no differences between programs (*p* = 0.566, Cramér’s V = 0.08). As in previous variables, public health students reported the lowest rate 63.6%. Nonetheless, interest in participating in AI-related training seminars differed significantly across programs (*p* = 0.021, Cramér’s V = 0.18). Nursing (77.0%) and Physiotherapy (75.5%) students expressed the highest interest, followed by Pharmacy (71.6%), whereas public health students barely reached a positive majority (52.3%) to participate in AI training workshops.

The final question was about participants’ attitude towards the ability of AI to substitute them for their future professional work. A positive response was reported the lowest amongst physiotherapy students (10.2%), which was almost doubled by the second-placed nursing students (19.0%). Pharmacy and public health students reported the highest belief that they could be replaced by AI developments (25.7% and 27.3%, respectively).

In the context of a high percentage of students using AI in both their academic and daily life, 38.9% reported being comfortable (38.9%) or very comfortable (20.4%) working with AI tools for knowledge acquisition, 37.4% were neutral, while only a small minority expressed negative attitudes (2.3% uncomfortable and 0.7% very uncomfortable). When asked on the perceived impact of AI on classroom activities, the majority of students reported a positive impact (74.8%), followed by a 21.2% perceiving no impact, and only 4.0% believing that AI had a negative effect.

**Fig. 3:**
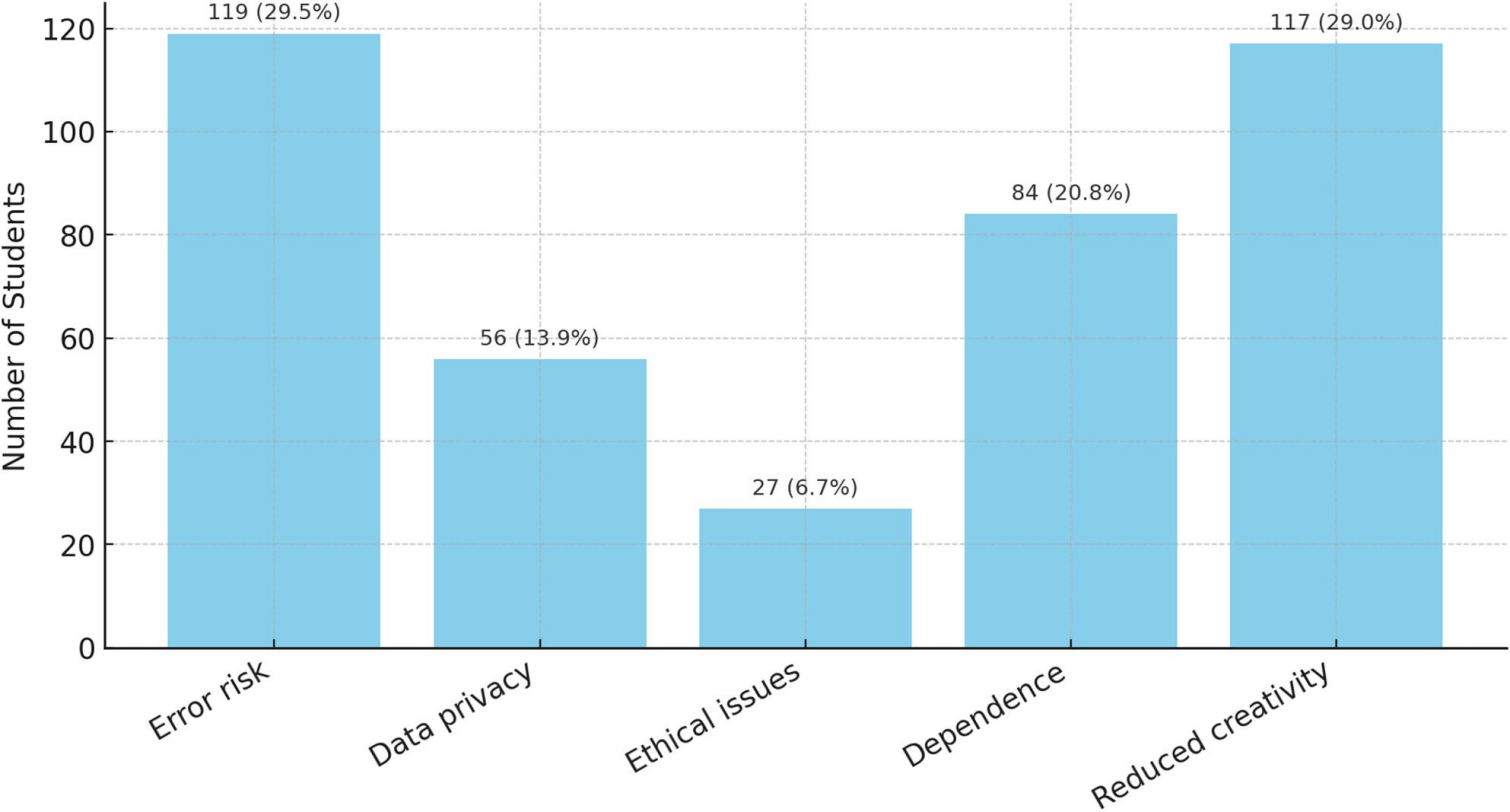
Concerns regarding the use of AI in education

When assessing the students’ concerns on the use of AI in education, the most common was error risk (29.5%) and reduced creativity (29.0%), followed by dependence on AI (20.8%), data privacy (13.9%) and ethical concerns (6.7%). Figure 3 visualizes these exact concerns.

## Discussion

### Student knowledge and attitudes

This study provides insights into how entry level healthcare students in a developing upper-middle-income country like Kosovo perceive and engage with generative AI in their professional education. To our knowledge, this is the first study examining generative AI-related knowledge and self-reported understanding and perceived institutional readiness among healthcare students in Kosovo, contributing data from a developing Balkan context that is underrepresented in current literature. Findings highlight a widespread exposure to AI tools like ChatGPT, but training opportunities and institutional support varied across programs. Besides the overall attitudes being positive, notable differences were observed between disciplines, this way highlighting differences across study programs in AI-related experiences and perceptions. Results deriving from this study contribute to the growing international evidence base on AI incorporation in and interaction with health education, while offering a valuable perspective from the perspective of a developing country. When interpreting these findings in relation to the TAM, the generally positive attitudes of students toward generative AI and its perceived usefulness for learning may be consistent with key constructs such as perceived usefulness and ease of use, although these were not directly measured. Reported easy use and willingness to engage with these tools may suggest patterns aligned with behavioural intention. These findings may reflect both individual perceptions and program-level educational environments, and are broadly consistent with previous studies applying TAM in educational contexts, where perceived usefulness and ease of use are associated with AI adoption [16, 22]. From a Connectivist perspective, the frequent use of ChatGPT and similar platforms may suggest that students may engage in forms of networked knowledge construction and retrieval, with AI tools potentially functioning as nodes within broader knowledgesystems. In this context, generative AI can be viewed as both educational technology and a knowledge-management medium. knowledge-management medium. However, these interpretations are exploratory, reflecting self-reported perceptions within a descriptive cross-sectional design. Accordingly, while the findings may be interpreted in relation to TAM and connectivist principles, they do not constitute a formal test of these frameworks. From a knowledge management perspective, the results may suggest a potential role of generative AI in supporting both individual learning and broader knowledge-sharing environments. These theoretical frameworks are used to support interpretation of findings rather than to imply direct measurement or causal relationships.Findings from this study, even though indicating a comparable baseline familiarity with AI from students of all educational programs, provide particular disparities in terms of exposure to AI tools, structured training and institutional support. Students of public health program seemed to be significantly less informed by their teachers on AI-assisted teaching material use (*p* < 0.01), having received less formal training on AI integration on teaching (*p* < 0.05) and reported lower institutional support (*p* < 0.05). There is not enough evidence that institutional affiliation was related to this outcome, but it could perhaps be explained with public health students being younger than their counterparts (*p* = 0.004). Differences between programs should be interpreted with caution, as potential confounding factors such as age and study year were not directly analyzed in relation to outcomes. A recent study by Shihehgar et al. [18] also observed that seniors and those from higher degrees exhibited more favourable outcomes in comparison to their less experienced peers (particularly on education and AI). Consistent with Shishehgar et al. [18], findings may be partially explained by differences in academic exposure across study years, suggesting that greater experience within the educational environment may be associated with increased AI readiness. This way results from our study suggest that program-specific contexts and academic seniority are important factors shaping AI adoption. Recognizing this diversity is essential for designing tailored interventions that address not only individual student needs but also structural differences between programs and the specificities of each program.

### Institutional readiness and inequities

With respect to the institutional support for providing AI tools and other means for their appropriate integration in academia, the vast majority of students (75.8%) reported for none. However, this finding should be interpreted with caution, as individual access to AI tools was not assessed and may influence students’ perceptions. To some extent, this may reflect broader structural constraints, as many AI tools require technological resources and faculty expertise that may be limited in low-resource settings [23]. Findings that 75.8% of students perceived a lack of institutional support (Table 2) highlights a significant gap in formal AI integration. While the study did not control for private access or paid subscriptions (which could influence individual usage patterns) the high overall adoption of free tools like ChatGPT (84.2%) suggests that students are independently accessing AI. Therefore, the perceived lack of support may reflect a deficit in institutional guidance, ethical frameworks, and curricular integration rather than a lack of physical or digital access to the technology itself. However, in the ever-evolving education digitalization of health education through curricular interventions may be important to maintain qualitaty and competitiveness. Similar calls for institutional adaptability and technology-driven reform have been noted in higher education globally, where online and digital technologies are positioned as crucial for resilience and future preparedness [24]. Topics like DHL and AI education must be also officially integrated within healthcare curricula as mechanisms allowing future health professionals to be equipped with the necessary digital skills to navigate, interpret, and act with both confidence and efficacy [6]. Evidence based practice is one of the better education approaches that has been shown to significantly enhance the quality of teaching in entry level medical education programs [6], and the AI may serve as a useful facilitator of its faster and smoother inclusion. Our findings suggest the potential value of embedding structured AI-related knowledge training within healthcare curricula. In this context, curriculum integration refers to the systematic inclusion of AI in formal teaching, including dedicated AI literacy modules, practical workshops on responsible use, and integration within existing courses such as evidence-based practice and research methodology [23]. Beyond general positive attitudes toward AI, this study provides additional insights into program-level disparities in training opportunities and perceived institutional support, highlighting structural gaps that may influence equitable AI integration in healthcare education. Institutional support therefore represents a key enabling factor, as without adequate infrastructure, faculty training, and policy alignment, student enthusiasm for AI cannot translate into sustainable curricular innovation. This particular finding echoes global calls for aligning digital strategies with pedagogical goals, particularly in low-resource environments.

### Curricular and policy implications

It is important to note that young generations of students are nowadays equipped with valuable knowledge on AI and skills to navigate within the vast possibilities offered to them. Such findings have been highlighted by a recent study by Ahsan [23] while suggesting that most initiatives remain at very early stages with limited data on the best practices to follow. The rather low level of AI integration within curricula in our cohort (as reported by students) is a fact to be acknowledged. It is understandable that teachers from some particular programs like physiotherapy might find the usefulness of AI less compelling with others, it does not mean that AI and its algorithmic means cannot be integrated into the diagnostic process and help design individualized intervention plans. However, the fact that less than one third of teachers inform their students on their use of AI-assisted teaching material is an issue to be addressed. Rapid AI developments, combined with limited teacher training, make this a fragile pathway to navigate. Yet, a probably crucial component of this process would be a responsible integration of AI including ethical training, beyond the mere technical proficiency. Volatile issues like plagiarism, algorithmic bias, data privacy, and clinical accountability remain underexplored topics by our participating students and warrant explicit curricular attention. As observed, the major concerns regarding the use of AI in education started with error risk (29.5%), reduced creativity (29.0%), dependence (20.8%), data privacy (13.9%) and ended with ethical issues (6.7%). In this context, preparing future healthcare experts to act as ethical stewards of AI will be crucial in preserving humanistic care in the digital era.

Other notable findings deriving from this study were the generally positive attitudes towards the potential benefits from the AI integration in curricula, that was observed amongst participants. 74% of participants perceived a positive impact of AI integration into their teaching, 91.4% were positive that AI facilitates access to information, and 77.2% acknowledged the importance of integrating AI into curricula. Furthermore, the belief that students should develop AI-based knowledge and skills (70.5%) together with the interest of 71.2% of participants to follow AI training or workshops, suggests the extent of their trust in the capabilities and necessities that AI holds for their future developments. The fact that this interest to learn about AI in their respective healthcare field is almost double to what Rjoop and colleagues [25] previously reported in their comprehensive study (46.2% in Jordan students) is a positive sign that should be seriously taken in consideration when developing and updating the existing education curricula in our country. Beyond the national context, such findings contribute to the global discourse of AI education. Unprepared educators, limited digital infrastructure and AI tools risk enhancing educational disparities both within Kosovo and worldwide. Even though previously mentioned the unreadiness of academic staff (neither equipped nor trained) with new applications or technologies [26], positioning data from an upper-middle-income European country like Kosovo contributes to balancing a literature still dominated by high-income countries, and for ensuring that AI integration in health education does not widen existing inequities but instead supports more inclusive capacity-building. Our findings underscore the need for structured AI literacy modules and digital pedagogy reforms across higher education curricula, ensuring equitable access to AI in learning and teaching. Without such measures, AI adoption risks deepening educational inequities.

These findings suggest that the biological gender of participants does not play a major role in shaping students’ knowledge, experiences, or attitudes toward AI in entry level health education. Besides a small difference in reported institutional provision of AI tools, with men indicating slightly higher access, no other significant gender disparities could be observed across other variables. This in fact aligns with previous studies indicating that attitudes toward digital health and AI adoption are not influenced by biological gender [7, 27, 28], in comparison to other studies claiming the opposite with men demonstrating higher usage of AI [29] or being more likely to believe that AI will improve diagnostic accuracy [30].

When asked the students on how they perceived the possibility for AI-means to substitute them in a near future, it was interesting to see that physiotherapy students expressed the least belief in this possibility, whereas public health students reported the highest perceived likelihood of being replaced (27.3%). This is to some extent understandable due to the nature of the work conducted by all of them, particularly with physiotherapy work typically characterized with a more personalized approach. However, the overall low number of respondents (21.2%) seeing AI as a threat is another positive point for its great potential in academia.

### Limitations

Even though conducting this study to the best of our knowledge and capabilities, certain limitations prevail. One important limitation to mention is the recruitment of younger participants from the public health program, which is supported by a Cramér’s V result of differences between study programs - study year participation to be 0.49. Such outcome suggests a large effect size, which considering the higher experience in academia and other issues related to it (e.g. AI), students from second and third year might have had different knowledge, experiences and opinions on the role of AI into entry level healthcare education. One other limitation was the modest internal consistency of the scale in the full sample (α = 0.61), which required item-level rather than composite analyses. While internal consistency was modest, the use of item-level analyses strengthens validity and underscores the need for further instrument development in cross-cultural higher education contexts. A final potential limitation might be the use of self-reported measures, a method which even though effective and practical, may still not fully capture the actual AI knowledge or competencies of the targeted population.

Furthermore, the study did not capture qualitative differences in how students used AI tools (e.g., passive retrieval vs. interactive learning), which may have important implications for interpreting AI engagement. Finally, while the study identifies high rates of AI adoption, the survey did not differentiate between “passive” use (e.g., direct output generation) and “interactive” pedagogical use (e.g., dialogue or concept testing). Since nearly 30% of students expressed concerns regarding a loss of creativity, future research should include qualitative methods to explore how these different modes of engagement impact the long-term critical thinking skills. Despite limitations, the present findings contribute to the limited evidence on generative AI and knowledge management in health education from developing settings and under-represented populations.

## Conclusion

To conclude, this study provides unique data on students self-rated knowledge, use and perspectives on AI’s integration in education curricula. It highlights the need for an organized approach to integrate AI within higher education curricula, particularly in developing countries. Higher self-reported AI knowledge, knowledge on the levels of AI integration in curricula and the importance and benefits from it are to be praised. Such outcomes provide a promising background needed for future developments on the field. However, significant gaps persist in both formal institutional support and equitable access to resources. This positive climate may support efforts by higher education institutions to embed AI-related knowledge within curricula and faculty development across disciplines, ensuring inclusive and equitable digital education in developing countries.

## Data Availability

Data are available from the corresponding author on reasonable request.

## Abbreviations

AI: Artificial Intelligence
CI: Confidence Interval
DHL: Digital Health Literacy
EBP: Evidence-Based Practice
N: Number
SD: Standard Deviation
TAM: Technology Acceptance Model
Y: Year
